# Effects of KTP Laser Bleaching on Traumatized Tooth Enamel

**DOI:** 10.5812/traumamon.18168

**Published:** 2014-03-18

**Authors:** Jun-Ichiro Kinoshita, Hamid Jafarzadeh, Atsufumi Manabe, Miki Nozawa, Tokiko Uchida, Paul Vincent Abbott

**Affiliations:** 1 Department of Esthetic Dentistry, School of Dentistry, Showa University, Tokyo, Japan; 2 Dental Research Center, Department of Endodontics, Faculty of Dentistry, Mashhad University of Medical Sciences, Mashhad, IR Iran; 3 Department of Clinical Cariology and Endodontology, School of Dentistry, Showa University, Tokyo, Japan; 4 School of Dentistry, University of Western Australia, Perth, Australia

**Keywords:** Lasers, Solid-State, Tooth Bleaching, Tooth Discoloration, Tooth Injuries

## Abstract

**Background:**

Bleaching of traumatized discolored teeth is considered an important issue in esthetic dentistry. Various methods have been introduced for bleaching, some of which may have adverse effects on soft or hard tissues of the tooth.

**Objectives:**

The objective of the study was to evaluate the effects of KTP laser bleaching on enamel of traumatized teeth.

**Materials and Methods:**

A square of 36 mm^2^ was chosen on the labial surface of 32 extracted teeth. The corners were drilled in order to indicate the location precisely. The shade of each sample was recorded and the teeth were divided into four groups, each with eight teeth: group A (Smartbleach with KTP laser for 30 seconds), group B (Smartbleach with G-Light for 5 minutes), group C (only Smartbleach for 10 minutes) and group D (control group with no bleaching). After one session of bleaching, shade assessment was performed again. In another experiment on nine teeth, Smartbleach with KTP laser was used for 150 seconds to 1500 seconds. The samples were critically processed and observed by using scanning electron microscope (SEM) to assess enamel damage. Data was statistically analyzed using Kruskal-Wallis test (confidence interval level were set at 95%).

**Results:**

Smartbleach was highly effective when used with KTP laser (P = 0.0419). Enamel damage was observed after frequent KTP bleaching and 750 seconds of KTP bleaching was recognized as the limit by morphological evaluation via SEM, indicating a major difference between under and over 750 seconds of bleaching.

**Conclusions:**

Application of KTP laser may increase the bleaching effect of Smartbleach, nevertheless it may cause some enamel damage.

## 1. Background

Tooth discoloration is considered a complication in traumatized teeth. Dentists believe that bleaching these types of teeth is an important part of their profession (1, 2). This type of treatment is the most commonly requested cosmetic service in most dental offices (2). One technique recommended for external bleaching involves laser application. Two types of lasers have been routinely employed for this purpose: the argon and the carbon dioxide (CO_2_) laser. These lasers can be targeted at the stained molecules by catalysts which split hydrogen peroxide to create oxygen and water (1). There is also another type of laser, called KTP laser (potassium-titanyl-phosphate), which is becoming popular in Europe and Japan (3-5). The KTP laser, a type of Nd: YAG laser, is practical for external bleaching. As the laser beam from the Nd: YAG laser is 1064 nm in wavelength. KTP wavelength is 532 nm, which is comparably just half the wavelength of the Nd: YAG laser. KTP laser has similar characteristics to Nd: YAG. While it has a few more unique characteristics (6); the green visible light of KTP laser is absorbed well by melanin and hemoglobin (7-9), but it is not absorbed well in water (10). This feature allows the KTP laser to penetrate dentin with less damage, since water is the major component of dentin. KTP lasers have high energy photons which are well-known for having shorter wavelength allowing chemical and photodynamic reactions to progress efficiently with less damage to both hard and soft tissues of dental pulp (11-15). Zhang points out that the KTP laser is capable of producing more significant teeth whitening than LED or diode laser techniques (15). However, it may have some adverse effects on tooth structure.

## 2. Objectives

Literature on the current subject is scant. In recent years, there is increasing demand for bleaching of traumatized teeth. Thus, the aims of this study were to evaluate the whitening effect of KTP laser on bleaching with Smartbleach whitening system (High-Tech Laser, Heizele, Belgium) and also to evaluate the adverse effects on tooth enamel.

## 3. Materials and Methods

The research was approved by the Ethical Committee of the Showa University. All the teeth samples were saturated in saline for two hours immediately after extraction. Then for the purpose of disinfection, they were placed in Germitor (0.025 w/v % benzalkonium chloride) solution.

### 3.1. Color Assessment

Thirty two non-carious extracted teeth were selected. A square of 36 mm^2^ was chosen on the labial enamel surfaces of each tooth where there were no dental caries. The corners of the square were drilled with a 0.3 mm round bur (Dentsply/Maillefer, Tulsa, Okla., the USA), in order to precisely identify the location of the experimental site. The teeth were saturated in saline at 37˚C for 10 days and then the shade of each sample at the intersection point of virtual diagonal lines of the square was recorded. The assessment of color was performed by using a ShadeEye (Shofu Inc., Kyoto, Japan) (instrumental assessment) along with a shade-guide visual assessment, VITAPAN Classical (Vita Zahnfabric, Bad Sackingen, Germany). The teeth were divided into four groups of eight teeth each. Group A received the Smartbleach with KTP laser (SmartLite, DECA, Florence, Italy) (one Watt power) for 30 seconds according to the manufacturer’s instructions. Group B received the Smartbleach with G-Light (Prima Ⅱ, GC, Tokyo, Japan) for five minutes. Group C received only the Smartbleach for 10 minutes and Group D the control group, received no bleaching. It is noteworthy to mention that G-light is a high powered LED lighting device which is useful for tooth whitening. Its wide range of wavelength - from blue (465 ± 10 nm) to purple (400 ± 10 nm) - can be used with almost all whitening materials.

The duration of irradiation with the laser and the light followed the manufacturers’ instructions. Distance of irradiation was set at 10 mm from the handpiece which was slowly moved for small spots over the tooth (7 mm diameter, which was the approximate width of tooth crowns). One session after the procedure, shade assessment was performed again by using ShadeEye and VITAPAN Classical. ΔE was calculated using CIE1976 L*a*b System; a system for assessing the difference between two colors where ΔE represents the "distance" between two colors. ΔE of 1.9 is the smallest color difference can be discriminated by human eye, therefore, any ΔE less than 1.9 is not observable whilst ΔE greater than 1.9 is perceptible (16). Each standard VITAPAN Classical shade guide was arranged based on the color degree, using ShadeEye ( B1 was the lightest color). Data was statistically analyzed using the Kruskal-Wallis test.

### 3.2. Assessment of the Bleached Enamel Morphology

Nine extracted anterior teeth were stored in saline for 10 days. Eight of them were used as experimental teeth and one as the control. The Smartbleach system with a KTP laser was used for 150, 300, 450, 600, 750, 900, 1200 and 1500 seconds on one tooth for each time interval. All samples were then saturated in ethylenediaminetetraacetic acid (EDTA) and dried using a drying machine, platinum coated as the traditional way and observed using SEM (Hitachi S4700, Tokyo, Japan). The control tooth had no bleaching and no laser application. It was prepared and observed under scanning electron microscope (SEM) in the same manner as the experimental teeth.

## 4. Results

### 4.1. Color Evaluation

Visual assessment indicated that in all of the experimental groups, the shade changed for the better by more than three degrees. There was no visible change in the control teeth. The ΔE was 3.63 in group A, 2.67 in group B, 2.77 in group C and 1.00 in group D. There were significant differences between the three experimental groups (P = 0.0419, Tables 1 and 2). Tukey’s HSD showed that Smartbleach was the most effective when used with KTP laser.

**Table 1.** Quantitative Results of the First Part of the Study ^a,^ ^b^

	A	B	C
L* (Clear, Bright)	64.2 to 65.9	63.6 to 65.9	64.3 to 66.5
a* (Red, Green)	2.1 to 1.4	0.4 to 0.2	1.0 to 0.7
b* (Yellow, Blue)	11.1 to 10.4	10.6 to 10.7	11.2 to 9.8
**VITA shade**	A2 to A1	A2 to A1	A2 to A1
**VITA shade Change**	3 steps better	3 steps better	3 steps better
**Delta E**	3.637307	2.670206	2.774887

^a^ Group A, only SB; group B, SM with G-light; group C, SB with KTP.

^b^ Random samples were selected from the three groups. The results proved to be average for all three groups. Delta E showed that all the groups showed notable changes (above delta 1.9). The score for group D (control group) with no whitening procedure was considered to be delta 1.0.

**Table 2.** Enamel Morphology Evaluation After Bleaching

Exposure Time, s	Magnification	Study Result
**0**		
	10000X	Smooth enamel surface
	30000X	Intact enamel particles
**30**	-	No enamel damage
**150**	-	No enamel damage
**300**	-	No enamel damage
**450**	-	No enamel damage
**600**		
	10000X	Scarring of the enamel other parts of the surface were smoother than control group
	30000X	Intact enamel particles
**750**		
	10000X	Partial loss of the enamel surface
	30000X	Loss of the connection between enamel particles
**900 or 1500**		
	10000X	Enamel damage
	30000X	Loss of enamel particles from the surface

### 4.2. Morphological Evaluation of Bleached Enamel

**Figure 1.**
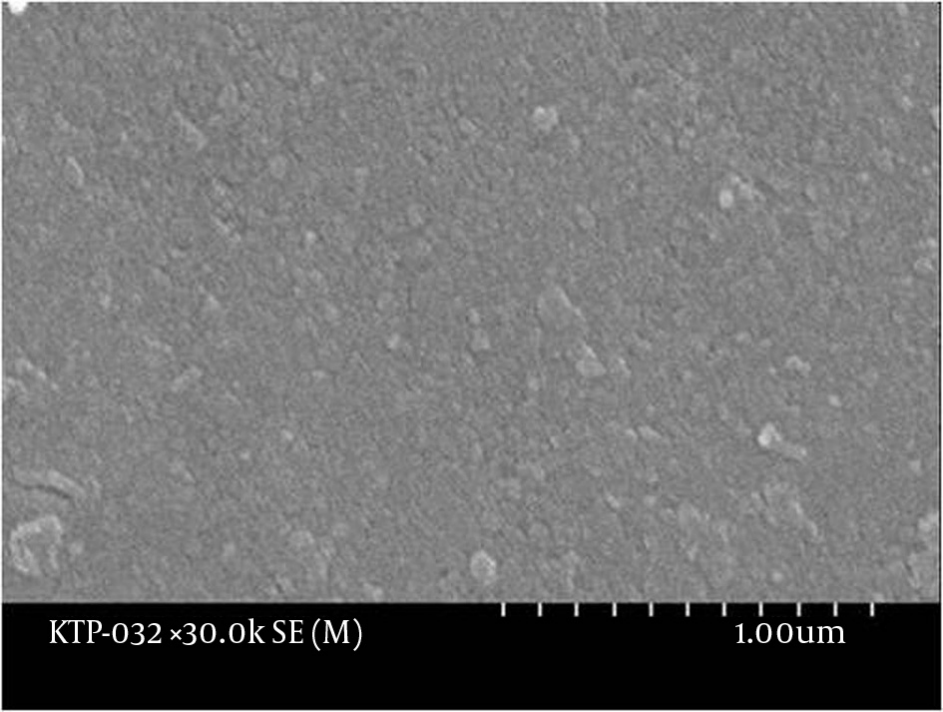
Scanning Electron Microscope Image of Control Sample Observed at 30000X Magnification Intact enamel particles can be observed.

**Figure 2.**
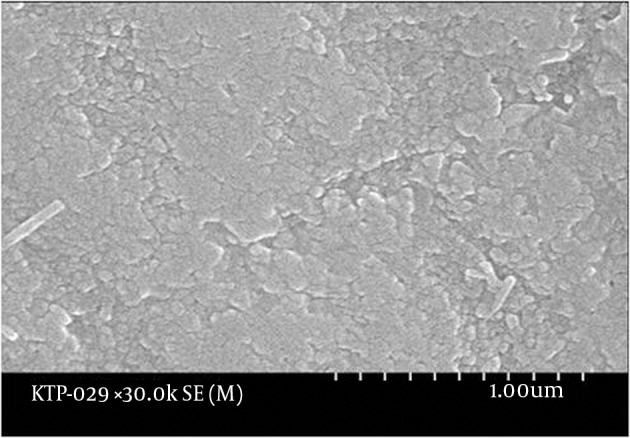
Scanning Electron Microscope Appearance of Enamel After 750 Seconds of KTP Laser Treatment at 30000X Magnification The eroded surface suggests the loss of contact between enamel particles.

**Figure 3.**
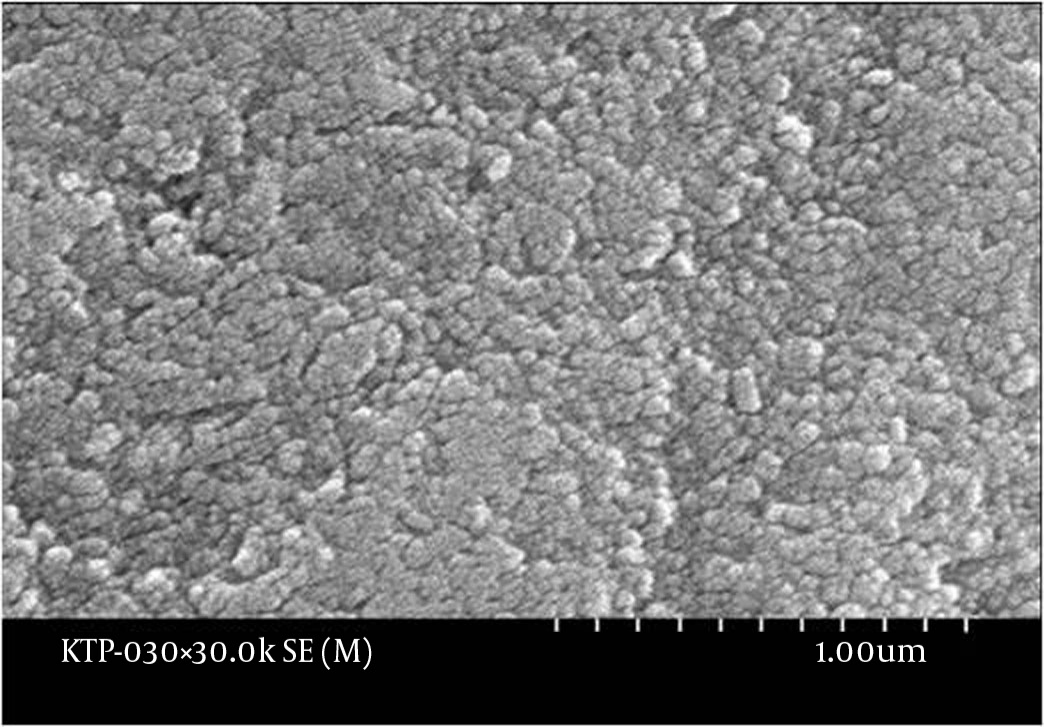
Scanning Electron Microscope Appearance of Enamel After 1500 Seconds of KTP Laser at 30000X Magnification Damage due to the laser exposure more than 750 seconds can be observed. Some enamel particles were lost from the surface. The “half-floating” particles appear to be removed if any mechanical force (e.g. tooth brushing) is exerted.

The enamel surface of the control teeth was smooth when observed at 10000X magnification. Intact enamel particles were also observed at 30000X magnification (Figure 1). The tooth exposed to 600 seconds of KTP laser showed scarring on the enamel at 10000X magnification, but it appeared to be the original damage caused mechanically and not chemically or thermally. Other parts of the surface were observed to be smoother in comparison with the control group. At 30000X magnification, intact enamel particles were observed. Teeth exposed to 30, 150, 300 and 450 seconds of bleaching and laser revealed no evident enamel damage when examined under SEM. The tooth exposed to bleaching and laser treatment for 750 seconds showed partial loss of the surface enamel at 10000X magnifications. At 30000X magnification, the eroded surface suggested no contact between the enamel particles (Figure 2). The tooth with 1500 seconds of exposure to bleaching and the laser showed enamel damage when observed at 10000X magnification. At 30000X magnification provided that a mechanical force was exerted (e.g. tooth brushing), enamel particles were lost from the surface (Figure 3). The tooth exposed to 900 seconds of bleaching and laser treatment showed similar damage.

## 5. Discussion

The dental bleaching mechanism varies depending on discoloration type. Bleaching materials affect organic structure of the hard tissues of the teeth and degrade them into lighter byproducts. However, this procedure can have some adverse effects including external root resorption, chemical burns, damage restoration, dental pulp and/or hard tissue damage (2, 16). To explain the photo-chemical reaction, the KTP laser tends to penetrate dentin more easily, because its wavelength is not well absorbed in water (as it is rich in dentin) and the gel penetrates through the outer enamel and into the dentin layer and gives the tooth surface a lighter reflection, which results in whiter teeth (1).

The Smartbleach system used in this study consisted of two materials, namely a transparent liquid and a red-purple powder. The liquid is 35% hydrogen peroxide which is slightly acidic. This concentration of hydrogen peroxide does not have strong potential energy as an oxidation agent if used alone, because its chemical reaction produces mainly the weaker oxide radical. The red-purple powder is alkaline and when mixed with the liquid, the mixture is buffered, producing a much stronger oxide radical. After being mixed, the gel becomes 27% hydrogen peroxide which has a glue-like consistency, helping to stay on the surface of the enamel. These features create a more efficient interaction between the large light-absorbing molecules in the teeth and the gel. This interaction breaks the chains of the large molecules, in a way that the ratio of the small molecules increases and the tooth surface becomes whiter. Hydrogen peroxide can become saturated in the dental hard tissues. Owing to its very small molecular weight, it penetrates into the organic substance in the crystals of hydroxyapatite. The KTP laser accelerates the reaction, resulting in brightening of the collagen color (6, 17-20).

In this study, experimental teeth bleaching showed remarkable effects. Group A had the highest ΔE, revealing that the combination of KTP laser and Smartbleach whitening system was the most effective. Nonetheless, enamel damage occurred with KTP bleaching performed for 750 seconds or more. Hence, in the status quo where there is no evaluation available on enamel damages by Smartbleach, it is recommended not to apply the Smartbleach gel with KTP irradiation for over 750 seconds to avoid breaking the enamel surface. SEM limitations such as the necessity of fitting samples into the microscope chamber should also be taken into consideration. Few studies have evaluated the effect of KTP laser on tooth structure. Tewfik et al. (11) deduced that KTP laser cannot change the permeability of the dentin covered by smear layer although SEM examination showed modifications to the surface of smear layer. All in all, more studies on not only the efficacy of KTP laser for bleaching of discolored teeth and comparing it with other types of lasers but also its possible impact on pulp tissue are required.
